# WEALTH DISPARITIES IN LATER LIFE: INSIGHTS AND IMPLICATIONS

**DOI:** 10.1093/geroni/igae098.0967

**Published:** 2024-12-31

**Authors:** Julie Miller

**Affiliations:** Chair

## Abstract

In the United States, wealth inequality is a stubborn and growing social and economic issue. Disparities in wealth are both products of, and contributors to, a range of pressing issues that directly impact financial wellbeing of individuals, households, and economies at large. By the time individuals reach older age, an individual’s financial wellbeing has generally compounded, making disparities in wealth experienced across sub-groups of older adults that much clearer. Considered in contexts of wealth transfer and financial capacity-building across generations, we also recognize that the implications of wealth disparities often reach across generations.

This symposium brings together leading scholars to share insights about wealth disparities in older age based on a range of identities and experiences. The first presentation focuses on wealth disparities among older LGBTQ+ adults, centering generational and social forces in the life events and experiences of their interactions with financial wellbeing. The second presentation focuses on the intersection of wills, wealth, and race, introducing context about estate planning and disparities in wealth transfer. The third and fourth presentations connect corollary issues with wealth disparities in later life. One presentation evaluates whether the sense of meaning and purpose in life mitigates these existing wealth disparities in later life, and the other presentation investigates how health and marital status shocks related to wealth disparities among older adults. Together, the four presentations introduce new perspectives about wealth inequality and wealth disparities in older age, and suggest implications for research, policy, and practice. Economics of Aging Interest Group Sponsored Symposium

